# Robustness to Noise in the Auditory System: A Distributed and Predictable Property

**DOI:** 10.1523/ENEURO.0043-21.2021

**Published:** 2021-03-17

**Authors:** S. Souffi, C. Lorenzi, C. Huetz, J.-M. Edeline

**Affiliations:** 1 Paris-Saclay Institute of Neuroscience (Neuro-PSI), Department Integrative and Computational Neuroscience, Unité Mixte de Recherche (UMR 9197) Centre National de la Recherche Scientifique Orsay 91405, France; 2 Université Paris-Saclay, Orsay Cedex 91405, France; 3 Laboratoire des Systèmes Perceptifs, Unité Mixte de Recherche (UMR 8248) Centre National de la Recherche Scientifique, Département d’Etudes Cognitives, Ecole Normale Supérieure, Université Paris Sciences et Lettres, Paris 75005, France

**Keywords:** auditory system, natural vocalizations, noise resistance, neuronal classification, noise-type sensitivity

## Abstract

Background noise strongly penalizes auditory perception of speech in humans or vocalizations in animals. Despite this, auditory neurons are still able to detect communications sounds against considerable levels of background noise. We collected neuronal recordings in cochlear nucleus (CN), inferior colliculus (IC), auditory thalamus, and primary and secondary auditory cortex in response to vocalizations presented either against a stationary or a chorus noise in anesthetized guinea pigs at three signal-to-noise ratios (SNRs; −10, 0, and 10 dB). We provide evidence that, at each level of the auditory system, five behaviors in noise exist within a continuum, from neurons with high-fidelity representations of the signal, mostly found in IC and thalamus, to neurons with high-fidelity representations of the noise, mostly found in CN for the stationary noise and in similar proportions in each structure for the chorus noise. The two cortical areas displayed fewer robust responses than the IC and thalamus. Furthermore, between 21% and 72% of the neurons (depending on the structure) switch categories from one background noise to another, even if the initial assignment of these neurons to a category was confirmed by a severe bootstrap procedure. Importantly, supervised learning pointed out that assigning a recording to one of the five categories can be predicted up to a maximum of 70% based on both the response to signal alone and noise alone.

## Significance Statement

In daily situations, humans and animals are faced with various background noises in which they have to detect behaviorally salient signals. Noise resistance is often viewed as an emergent property of cortical networks, but only a few studies have characterized the relative contribution of cortical and subcortical neurons. Our results demonstrate that the neuronal resistance to noise is distributed along the auditory system with a more important fraction of robust neurons in subcortical structures compared with auditory cortex, and is relatively well predictable based on the responses to the signal alone and the noise alone. Our results also suggest that noise-invariant representations of communication sounds coexist with accurate noise representations, which are detected as early as the cochlear nucleus (CN).

## Introduction

In natural conditions, speech (in humans) and communication sounds (in animals) usually co-occur with many other competing acoustic signals. During evolution, the auditory system has developed strategies to extract these behaviorally important signals mixed up with substantial amounts of noise. Over the last decade, many studies performed on different species have reported that the responses of auditory cortex neurons are quite resistant to various types of noises, even at low signal-to-noise ratio (SNR; Narayan et al., 2007; Rabinowitz et al., 2013; Schneider and Woolley, 2013; Mesgarani et al., 2014; Ni et al., 2017; Beetz et al., 2018). Several hypotheses have been formulated to account for the high performance of auditory cortex neurons. For example, it was proposed that noise tolerance is correlated with adaptation to the stimulus statistics, potentially more pronounced at the cortical than at the subcortical level (Rabinowitz et al., 2013). A dynamic model of synaptic depression was also suggested as a potential mechanism for robust speech representation in the auditory cortex (Mesgarani et al., 2014). Alternatively, a simple feedforward inhibition circuit was viewed as a mechanism to explain background-invariant responses detected in the secondary auditory cortex (Schneider and Woolley, 2013).

A recent study (Ni et al., 2017) reported that auditory cortex neurons can be assigned to categories depending on their robustness to noise. By testing the responses to conspecific vocalizations at different SNRs, this study described four types of response categories (robust, balanced, insensitive, and brittle) in the marmoset primary auditory cortex (A1), and pointed out that depending on the background noise, two-thirds of A1 neurons exhibit different response classes (Ni et al., 2017).

The present study aimed at determining whether the subcortical auditory structures display similar proportions of these four categories and whether the noise-type sensitivity is already present at the subcortical level. We used the same methodology as in Ni et al. (2017) to assign each recording to a given response class: the extraction index (EI; initially defined by Schneider and Woolley, 2013) was computed at three SNRs (+10, 0, and −10 dB) and an unsupervised clustering approach (the K-means algorithm) revealed groups of EI profiles in a given background noise.

We performed this clustering approach in the cochlear nucleus (CN), inferior colliculus (IC), auditory thalamus, primary and secondary auditory cortex using two types of masking noise, a stationary or a chorus noise composed of a mixture of conspecific vocalizations. We renamed two neuronal behaviors in noise for recognizing equivalent roles to stimulus-like neuronal responses (i.e., the signal-like and the masker-like responses) since in ethological conditions, both could play an important functional role.

The categories range from signal-like responses (equivalent to the “robust” neurons of Ni et al., 2017) showing a high-fidelity representation of the signal, to masker-like responses showing a high-fidelity representation of the noise (equivalent to the “brittle” neurons of Ni et al., 2017), with two intermediary categories, one showing no preference either for the signal or for the noise named insensitive, and the other characterized by the highest sensitivity to the SNR named balanced. To minimize the intra-category distances, we also added a new category, called signal-dominated, which corresponds to an attenuated version of the signal-like responses.

Here, we present evidence that the categories initially described by Ni et al. (2017) in the A1 do exist at each stage of the auditory system, from CN to secondary auditory cortex. From a continuum of EI values, we imposed a clustering and revealed that each category was represented at each relay of the auditory system in different proportions, depending on the auditory structure and the type of the masking noise. Signal-like and signal-dominated responses were in higher proportions in IC and thalamus in both noises. Masker-like responses were found mostly in the CN in stationary noise but in similar proportions in each structure in chorus noise. Interestingly, the proportion of balanced responses decreased as one ascends in the auditory system suggesting a decreased sensitivity to SNR at the cortical level. The noise-type sensitivity, that is the ability to switch category from a given background noise to another, exists at each level of the auditory system. Using a supervised learning approach with descriptors extracted from the responses to the original vocalizations alone (signal) and to the maskers alone, we provide evidence that the assignment to a given category is relatively well predicted in both types of noise.

## Materials and Methods

Most of the Methods are similar to those described in a previous study (Souffi et al., 2020).

### Subjects

These experiments were performed under the national license A-91-557 (project 2014-25, authorization 05,202.02) and using the procedures N° 32-2011 and 34-2012 validated by the Ethic committee N°59 (CEEA Paris Centre et Sud). All surgical procedures were performed in accordance with the guidelines established by the European Communities Council Directive (2010/63/EU Council Directive Decree).

Extracellular recordings were obtained from 47 adult pigmented guinea pigs (aged 3–16 months, 36 males, 11 females) at five different levels of the auditory system: the CN, the IC, the medial geniculate body (MGB), the primary (A1) and secondary [area ventrorostral belt (VRB)] auditory cortex. Animals, weighting from 515 to 1100 g (mean 856 g), came from our own colony housed in a humidity (50–55%)-controlled and temperature (22–24°C)-controlled facility on a 12/12 h light/dark cycle (light on at 7:30 A.M.) with free access to food and water.

Two days before the experiment, the animal’s pure-tone audiogram was determined by testing auditory brainstem responses (ABRs) under isoflurane anesthesia (2.5%) as described in Gourévitch et al. (2009). A software (RTLab, EchodiaFrance) allowed averaging 500 responses during the presentation of nine pure-tone frequencies (between 0.5 and 32 kHz) delivered by a speaker (Knowles Electronics) placed in the animal right ear canal. The auditory threshold of each ABR was the lowest intensity where a small ABR wave can still be detected (usually Wave III). For each frequency, the threshold was determined by gradually decreasing the sound intensity (from 80-dB SPL down to −10-dB SPL). All animals used in this study had normal pure-tone audiograms (Gourévitch et al., 2009; Gourévitch and Edeline, 2011).

### Surgical procedures

All animals were anesthetized by an initial injection of urethane (1.2 g/kg, i.p.) supplemented by additional doses of urethane (0.5 g/kg, i.p.) when reflex movements were observed after pinching the hind paw (usually two to four times during the recording session). A single dose of atropine sulfate (0.06 mg/kg, s.c.) was given to reduce bronchial secretions and a small dose of buprenorphine was administrated (0.05 mg/kg, s.c.) as urethane has no antalgic properties. After placing the animal in a stereotaxic frame, a craniotomy was performed and a local anesthetic (xylocain 2%) was liberally injected in the wound.

For auditory cortex recordings (areas A1 and VRB), a craniotomy was performed above the left temporal cortex. The dura above the auditory cortex was removed under binocular control and the cerebrospinal fluid was drained through the cisterna to prevent the occurrence of edema. For the recordings in MGB, a craniotomy was performed above the most posterior part of the MGB (8 mm posterior to bregma) to reach the left auditory thalamus at a location where the MGB is mainly composed of its ventral, tonotopic, part (Redies et al., 1989; Edeline et al., 1999; Anderson et al., 2007; Wallace et al., 2007). For IC recordings, a craniotomy was performed above the left IC and portions of the cortex were aspirated to expose the surface of the left IC (Malmierca et al., 1995, 1996; Rees et al., 1997). For CN recordings, after opening the skull above the left cerebellum, portions of the cerebellum were aspirated to expose the surface of the left CN (Paraouty et al., 2018).

After all surgeries, a pedestal in dental acrylic cement was built to allow an atraumatic fixation of the animal’s head during the recording session. The stereotaxic frame supporting the animal was placed in a sound-attenuating chamber (IAC, model AC1). At the end of the recording session, a lethal dose of Exagon (pentobarbital >200 mg/kg, i.p.) was administered to the animal.

### Recording procedures

Data from multiunit recordings were collected in five auditory structures, the non-primary cortical area VRB, the primary cortical area A1, the MGB, the IC, and the CN (see Table 1). In a given animal, neuronal recordings were only collected in one auditory structure.

**Table 1:** Summary of the number of animals and number of selected recordings in each structure.

	CN	Lemniscal pathway			Non-lemniscal pathway	Total
		CNIC	MGv	A1	VRB	
Number of animals	10	11	10	11	5	47
Number of recordings tested	672	478	448	544	192	2334
Six EI values (for the six SNRs)	617	433	343	488	184	2065
One of the six EI values significantly higher than the EI_Surrogate_	428	374	230	349	137	1518
Selection type						
(a) Significant response to at least one vocalization and/or significant TFRP	401	350	210	279	109	1349
(b) Significant response to at least one vocalization and significant TFRP	389	339	198	261	80	1267

CN: cochlear nucleus, CNIC: central nucleus of inferior colliculus, MGv: ventral part of the MGB, A1: primary auditory cortex, VRB: ventrorostral belt.

A recording corresponds to a channel of a 16-channel electrode.

Cortical extracellular recordings were obtained from arrays of 16 tungsten electrodes (TDT, TuckerDavis Technologies; ø: 33 μm, <1 MΩ) composed of two rows of 8 electrodes separated by 1000 μm (350 μm between electrodes of the same row). A silver wire, used as ground, was inserted between the temporal bone and the dura matter on the contralateral side. The location of the A1 was estimated based on the pattern of vasculature observed in previous studies (Wallace et al., 2000; Gaucher et al., 2013; Gaucher and Edeline, 2015). The non-primary cortical area VRB was located ventral to A1 and distinguished because of its long latencies to pure tones (Rutkowski et al., 2002; Grimsley et al., 2012). For each experiment, the position of the electrode array was set in such a way that the two rows of eight electrodes sample neurons responding from low to high frequency when progressing in the rostro-caudal direction [see Gaucher et al. (2012; their Fig. 1) and Occelli et al. (2016; their Fig. 6*A*)].

**Figure 1.**
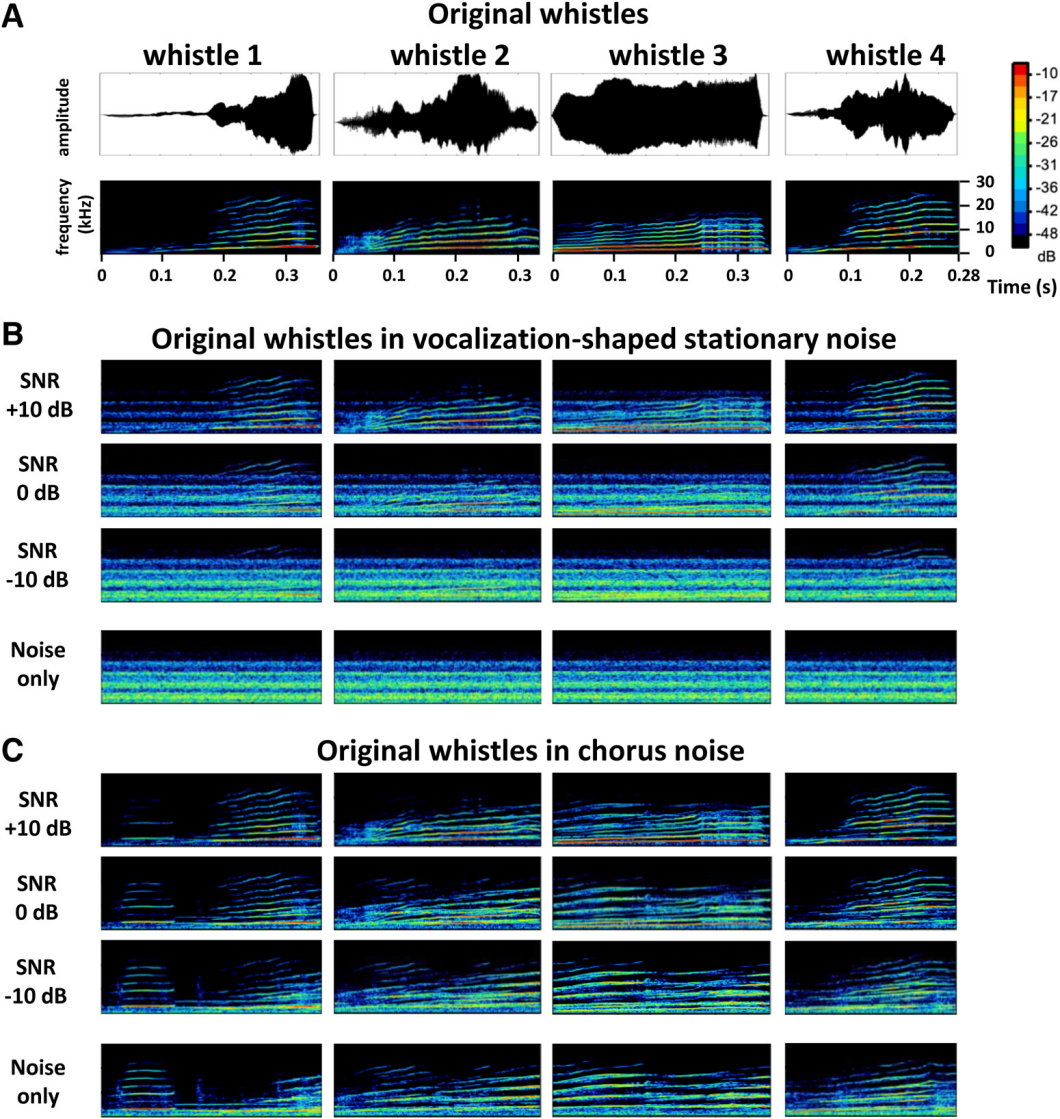
Original and noisy vocalizations. ***A***, Waveforms (top) and spectrograms (bottom) of the four original whistles used in this study. ***B***, ***C***, Spectrograms of the four whistles in stationary (***B***) and chorus (***C***) noise at three SNRs (+10, 0, and −10 dB, from top to bottom) and the noise only. The frequency range for all spectrograms is 0–30 kHz, and all spectrograms share the same color scale (covering a range of 50 dB).

In the MGB, IC and CN, the recordings were obtained using 16 channel multielectrode arrays (NeuroNexus) composed of one shank (10 mm) of 16 electrodes spaced by 110 μm and with conductive site areas of 177μm^2^. The electrodes were advanced vertically (for MGB and IC) or with a 40° angle (for CN) until evoked responses to pure tones could be detected on at least 10 electrodes.

All thalamic recordings were from the ventral part of MGB (see above surgical procedures) and all displayed latencies <9 ms. At the collicular level, we distinguished the lemniscal and non-lemniscal divisions of IC based on depth and on the latencies of pure tone responses. We excluded the most superficial recordings (until a depth of 1500 μm) and those exhibiting latency >= 20 ms in an attempt to select recordings from the central nucleus of the IC (CNIC). At the level of the CN, the recordings were collected from both the dorsal (DCN) and ventral (VCN) divisions, but based on the recording depth, we estimate that the DCN recordings were more numerous.

The raw signal was amplified 10,000 times (TDT Medusa). It was then processed by an RX5 multichannel data acquisition system (TDT). The signal collected from each electrode was filtered (610–10,000 Hz) to extract multiunit activity (MUA). The trigger level was set for each electrode to select the largest action potentials from the signal. On-line and off-line examination of the waveforms suggests that the MUA collected here was made of action potentials generated by a few neurons at the vicinity of the electrode. However, as we did not used tetrodes, the result of several clustering algorithms (Pouzat et al., 2004; Quiroga et al., 2004; Franke et al., 2015) based on spike waveform analyses were not reliable enough to isolate single units with good confidence. Although these are not direct proofs, the fact that the electrodes were of similar impedance (0.5–1 MΩ) and that the spike amplitudes had similar values (100–300 μV) for the cortical and the subcortical recordings, were two indications suggesting that the cluster recordings obtained in each structure included a similar number of neurons. Even if a similar number of neurons were recorded in the different structures, we cannot discard the possibility that the homogeneity of the multiunit recordings differs between structures. By collecting several hundreds of recordings in each structure, these potential differences in homogeneity should be attenuated in the present study.

### Acoustic stimuli

Acoustic stimuli were generated using MATLAB, transferred to a RP2.1-based sound delivery system (TDT) and sent to a Fostex speaker (FE87E). The speaker was placed at 2 cm from the guinea pig’s right ear (or left ear for CN recordings), a distance at which the speaker produced a flat spectrum (±3 dB) between 140 Hz and 36 kHz. Calibration of the speaker was made using noise and pure tones recorded by a Brüel & Kjær microphone 4133 coupled to a preamplifier B&K 2169 and a digital recorder Marantz PMD671. All the stimuli intensities were calculated as RMS.

The time-frequency response profiles (TFRPs) were determined using 129 pure-tones frequencies covering eight octaves (0.14–36 kHz) and presented at 75-dB SPL. The tones had a γ envelop given by $\gamma \left(t\right)=(\frac{t}{4})²e^{\frac{-t}{4}}$, where *t* is time in ms. At a given level, each frequency was repeated eight times at a rate of 2.35 Hz in pseudorandom order. The duration of these tones over half-peak amplitude was 15 ms, and the total duration of the tone was 50 ms, so there was no overlap between tones.

A set of four conspecific vocalizations was used to assess the neuronal responses to communication sounds. These vocalizations were recorded from animals of our colony. Pairs of animals were placed in the acoustic chamber and their vocalizations were recorded by a Brüel & Kjær microphone 4133 coupled to a preamplifier B&K 2169 and a digital recorder Marantz PMD671. A large set of whistle calls was loaded in the Audition software (Adobe Audition 3) and four representative examples of whistle were selected. As shown in Figure 1*A*, lower panels, despite the fact the maximal energy of the four selected whistles was in the same frequency range (typically between 4 and 26 kHz), these calls displayed slight differences in their spectrograms. In addition, their temporal (amplitude) envelopes clearly differed as shown by their waveforms (Fig. 1*A*, upper panels).

The four whistles were also presented in two frozen noises ranging from 10 to 24,000 Hz. To generate these noises, recordings were performed in the colony room where a large group of guinea pigs were housed (30–40; two to four animals/cage). Several 4 s of audio recordings were added up to generate the “chorus noise,” which power spectrum was computed using the Fourier transform. This spectrum was then used to shape the spectrum of a white Gaussian noise. The resulting vocalization-shaped stationary noise therefore matched the chorus-noise audio spectrum, which explains why some frequency bands were overrepresented in the vocalization-shaped stationary noise. Figure 1*B*,*C* displays the spectrograms of the four whistles in the vocalization-shaped stationary noise (Fig. 1*B*) and in the chorus noise (Fig. 1*C*) with a SNR of +10, 0, and −10 dB. The last spectrograms of these two figures represent the noises only.

### Experimental protocol

As inserting an array of 16 electrodes in a brain structure almost systematically induces a deformation of this structure, a 30-min recovering time lapse was allowed for the structure to return to its initial shape, then the array was slowly lowered. Tests based on measures of TFRPs were used to assess the quality of our recordings and to adjust electrodes’ depth. For auditory cortex recordings (A1 and VRB), the recording depth was 500–1000 μm, which corresponds to Layer III and the upper part of Layer IV according to Wallace and Palmer (2008). For thalamic recordings, the NeuroNexus probe was lowered ∼7 mm below pia before the first responses to pure tones were detected.

When a clear frequency tuning was obtained for at least 10 of the 16 electrodes, the stability of the tuning was assessed: we required that the recorded neurons displayed at least three successive similar TFRPs (each lasting 6 min) before starting the protocol. When the stability was satisfactory, the protocol was started by presenting the acoustic stimuli in the following order. We first presented the four whistles at 75-dB SPL in their original versions (in quiet), then the chorus and the vocalization-shaped stationary noises were presented at 75-dB SPL followed by the masked vocalizations presented against the chorus then against the vocalization-shaped stationary noise at 65-, 75-, and 85-dB SPL. Thus, the level of the original vocalizations was kept constant (75-dB SPL), and the noise level was increased (65-, 75-, and 85-dB SPL). In all cases, each vocalization was repeated 20 times. Presentation of this entire stimulus set lasted 45 min. The protocol was re-started either after moving the electrode arrays on the cortical map or after lowering the electrode at least by 300 μm for subcortical structures.

### Data analysis

All the analyses were performed on MATLAB 2019 (MathWorks).

### Quantification of responses to pure tones

The TFRP were obtained by constructing poststimulus time histograms for each frequency with 1-ms time bins. The firing rate evoked by each frequency was quantified by summing all the action potentials from the tone onset up to 100 ms after this onset. Thus, TFRP were matrices of 100 bins in abscissa (time) multiplied by 129 bins in ordinate (frequency). All TFRPs were smoothed with a uniform 5 × 5 bin window.

For each TFRP, the best frequency (BF) was defined as the frequency at which the highest firing rate was recorded. Peaks of significant response were automatically identified using the following procedure. A positive peak in the TFRP was defined as a contour of firing rate above the average level of the baseline activity plus six times the standard deviation of the baseline activity. Recordings without significant peak of responses or with inhibitory responses were excluded from the data analyses.

### Quantification of responses evoked by original vocalizations and noises alone

The responses to vocalizations were quantified using two parameters:

(1) The firing rate of the evoked response, which corresponds to the total number of action potentials occurring during the presentation of the stimulus.

(2) The trial-to-trial temporal reliability coefficient (CorrCoef) which quantifies the trial-to-trial reliability of the response over the 20 repetitions of the same stimulus. This index was computed for each vocalization: it corresponds to the normalized covariance between each pair of spike trains recorded at presentation of this vocalization and was calculated as follows:

$$CorrCoef=\frac{1}{\text{N(N-1)}}\sum_{i=1}^{N-1}\sum_{j=i+1}^{N}\frac{\sigma_{x_{i}}{}_{x_{j}}}{\sigma_{x_{i}}\sigma_{x_{j}}},$$

where *N* is the number of trials and *σx_i_x_j_*is the normalized covariance at zero lag between spike trains ξ and x_j_ where *i* and *j* are the trial numbers. Spike trains ξ and x_j_ were previously convolved with a 10-ms width Gaussian window. Based on computer simulations, we have previously shown that this CorrCoef index is not a function of the neurons’ firing rate (Gaucher et al., 2013).

We have computed the CorrCoef index with a Gaussian window ranging from 1 to 50 ms to determine whether the selection of a particular value for the Gaussian window influences the difference in CorrCoef mean values obtained in the different auditory structures. Based on the responses to the original vocalizations, we observed that the relative ranking between auditory structures remained unchanged whatever the size of the Gaussian window was. Therefore, we kept the value of 10 ms for the Gaussian window as it was used in several previous studies (Huetz et al., 2009; Gaucher et al., 2013; Gaucher and Edeline, 2015; Aushana et al., 2018; Souffi et al., 2020).

### Quantification of the EI

To evaluate the influence of noise on neural representation of vocalizations, we quantified the amount of vocalization encoded by neurons at a particular SNR level by calculating the EI adapted from a similar study in songbirds (Schneider and Woolley, 2013). This metric is based on the repetition-averaged peristimulus time histogram (PSTH) of neural response with a time bin of 4 ms. Different window bins of 1, 2, 4, 8, 16, and 32 ms were also evaluated, which yielded qualitatively similar results. In this manuscript, we only report results based on 4-ms time bins. Only the PSTH during the evoked activity is taken into account in this analysis.

EI was computed as follows:

$$EI=\frac{D_{n-snr}-D_{v-snr}}{D_{n-snr}-D_{v-snr}}$$

$$D_{n-snr}=1-\frac{\vec{P_{n}}\cdot \vec{P_{snr}}}{\left\|\vec{P_{n}}\right\|\left\|\vec{P_{snr}}\right\|},\;\;\;D_{v-snr}=1-\frac{\vec{P_{v}}\cdot \vec{P_{snr}}}{\left\|\vec{P_{v}}\right\|\left\|\vec{P_{snr}}\right\|},$$

where *D_n-snr_* is the distance between PSTH *_n_* of noise and PSTH _snr_ of vocalization at a particular SNR, whereas *D_v-snr_* is the distance between PSTH _v_ of pure vocalization and PSTH _snr_ of vocalization at a particular SNR. EI is bounded between −1 and 1: a positive value indicates that the neural response to noisy vocalization is more vocalization-like, and a negative value implies that the neural response is more noise-like. The EI profile for each recording was determined by computing EI at every SNR level. The normalized inner product was used to compute distance between _n_ or _v_ and _snr_, as shown in equation above.

To probe the response patterns of each neuron, we further implemented an exploratory analysis based on the calculated EI profiles as in Ni et al. (2017). By applying k-means clustering on the blended EI profiles from both noise conditions separately, we obtained, from a continuum of EI values, subgroups of EI profiles, which divided the neuronal population into clusters according to the similarity of their EI profiles. Similarity was quantified by Euclidean distance. The number of clusters was determined by the mean-squared error (MSE) of clustering, as in equation below,

MSE=1N∑i=1N(EIPcluster−i−EIPi¯)2,

where *N* is the number of neurons, EIP_i_ is the EI profile of a neuron, and _cluster-i_ is the mean EI profile of the cluster into which this neuron is categorized.

To determine a significance level for the EI of each neuron, we generated 100 false random spike trains which follow a Poisson law based on the firing rate values obtained for each stimulus (original and noisy vocalizations). For a given SNR and recording, we computed based on these false spike trains, 100 EI_Surrogate_ values and fixed a significance level corresponding to the mean plus two standard deviations. Using this criterion, we selected only the recordings with at least one of the six EI values significantly higher than the EI_Surrogate_.

### Bootstrap procedure

To estimate the variability of the EI index generated in assigning each recording to a particular category in particular noise, we used a bootstrap strategy for all the recordings, separately for the stationary and the chorus noise. Even in anesthetized animals, auditory cortex responses can show some variability. We suspected that in a given type of noise, a recording could change category because of the response variability and/or because the border between two clusters was very close, independently of the change in noise type.

From the 20 trials obtained for each stimulus during the experiment, we resampled randomly 20 trials (allowing repetitions) keeping the total number of trials the same as in the experimental data. For each resampled group of 20 trials, we recalculated both the PSTHs and the EI at each SNR then the K-means algorithm was used to define five clusters as in the experimental data. For each recording, this procedure was performed 100 times. Then, we reallocated each resampled data in the closest cluster compared with the original centroids of the experimental data to measure the percentage of changed categories relative to the original clustering.

### Classification using linear discriminant analysis (LDA)

In order to investigate whether the assignment of a given recording to a particular category can be predicted from the response characteristics obtained with pure tones and/or with the original vocalizations in quiet, and/or the noise responses alone, an automatic classification algorithm was applied. LDA classifiers were chosen among several linear supervised learning algorithms because of their slight higher classification performances when trained with all predictors (Statistics and Machine Learning Toolbox, MATLAB 2019). LDA is a statistical classifier that achieves a linear decision boundary based on the class scatter matrices. All classifiers used in this study were given identical parameters (same cost matrix, same cross-validation scheme), but were given different sets of predictors extracted from the data. A cost matrix was constructed to penalize the wrong assignment of all categories into the “insensitive” category that contained more recordings than the other categories (all costs were set to 1 except for the insensitive category where they were set to 2). Cross-validation was performed using a 5-fold validation scheme.

Fifteen LDA classifiers were built, trained and tested on the recordings considered as reliable with the bootstrap procedure (with a confidence interval ⩾95%) in both noises: 342 recordings were thus selected for the stationary and chorus noise (see Table 2, first line). For each recording and each type of noise (stationary or chorus), 12 predictors were available, extracted either from responses to pure tones, vocalizations, maskers or a combination of both signal and maskers. Each classifier has used a subset of predictors.

**Table 2:** Number of recordings reliably categorized both in stationary and in chorus noise using the bootstrap procedure and number of recordings sensitive to the type of noise within this population

	CN	Lemniscal pathway			Non-lemniscal pathway	Total
		CNIC	MGv	A1	VRB	
Number of recordings reliably categorized in the two noises	139	80	50	52	21	342
Number of recordings reliably categorized and noise-type sensitive	30	35	36	12	10	123
Number of recordings reliably categorized and no noise-type sensitive	109	45	14	40	11	219

### Descriptors used for the classifier

In total, 12 neuronal descriptors were extracted for each type of noise grouped in four types of descriptors: the TFRP, signal, masker, and signal-to-masker ratio descriptors. Three descriptors were extracted from the responses to pure tones (TFRP): the BF firing rate (in spikes/s), the bandwidth of tuning (in octave) and the response duration (in ms). From the responses to the signal alone (original vocalizations) and the maskers alone, two descriptors were extracted: the firing rate (in spikes/s) and the temporal reliability (or CorrCoef).

Three other masker descriptors were computed to have an estimation of the firing rate short-term adaptation to the masker. First, we computed the ratio between the firing rate taken at the time the signal should have occurred and the initial firing rate during the first 200 ms of the masker (FRm300 and FRm200). Second, we extracted the number of action potentials emitted during the first (initial) and last (final) 50 ms of the masker alone over a 564-ms period.

For the signal-to-masker ratios, the response to the maskers was extracted from the masker alone trials either from the initial firing rate (first 200 ms of the masker, FRm200), or from the firing rate taken at the time the signal should have occurred (i.e., for a mean duration of 300 ms, FRm300).

### Global quantification of category changes with mutual information (MI)

The MI allowed quantifying how many recordings change category from stationary noise to chorus noise based on either all recordings (Extended Data Fig. 5-1*A*) or only the reliable recordings (Extended Data Fig. 5-1*B*), independently of structure. For that, we built a matrix with five rows related to the five categories in stationary noise and five columns related to the becoming of each recording in chorus noise. From this matrix, the MI is given by Shannon’s formula (Shannon, 1948):

$$MI=\sum_{x,y}^{}p(x,y)\times \log_{2}(\frac{p(x,y)}{p(x)\times p(y)}),$$

where *x* and *y* are the rows and columns of the confusion matrix.

In a scenario where the categorization based on the responses in stationary noise do not carry information on the categorization based on the responses in chorus noise, assignment of each recording to a category is equivalent to chance level (here 0.20 because there were five different categories) and the MI would be close to zero. In the opposite case, when responses in stationary noise always fall in the same category when recorded with chorus noise, the confusion matrix would be diagonal and the MI would tend to log2(5) = 2.3 bits.

### Statistics

To assess the significance of the multiple comparisons (masking noise conditions: three levels; auditory structure: five levels), we used an ANOVA for multiple factors to analyze the whole dataset. *Post hoc* pairwise tests were performed between the noisy conditions (paired *t* tests) and between categories (Kruskal–Wallis tests). They were corrected for multiple comparisons using Bonferroni corrections and were considered as significant if their *p* value was below 0.05.

## Results

From a database of 2334 multiunit recordings collected in the five investigated auditory structures, several criteria were used to include each neuronal recording in our analyses (see Table 1). A recording had to show significant responses to pure tones (see Materials and Methods) and a significant evoked firing rate relative to spontaneous firing rate (200 ms before each original vocalization) in response to at least one of the four original vocalizations (Fig. 1*A* illustrates their waveforms and spectrograms). These four vocalizations were presented in quiet and embedded either in a vocalization-shaped stationary noise (Fig. 1*B*) or in a chorus noise (Fig. 1*C*) using three SNRs (+10, 0, and −10 dB). We selected recordings showing responses at the three SNRs both in stationary and chorus noise to derive systematically six EI values for each neuronal recording. The EI index quantifies to what extent the evoked response at a given SNR is similar to the response to vocalizations in quiet or to noise alone. To determine a significance level of the EI value, we computed an EI_Surrogate_ value for each recording (see Materials and Methods) and included only the recordings with at least one of the six EI values significantly higher than the EI_Surrogate_. Applying these criteria, we selected a total of 1267 recordings [Table 1, selection type (b)]: 389 in the CN, 339 in the CNIC, 198 in the ventral division of the auditory thalamus (MGv), 261 in the A1, and 80 in a secondary auditory cortical area (VRB).

### Chorus noise impacted more neuronal responses than stationary noise at each stage of the auditory system

**Figure 2.**
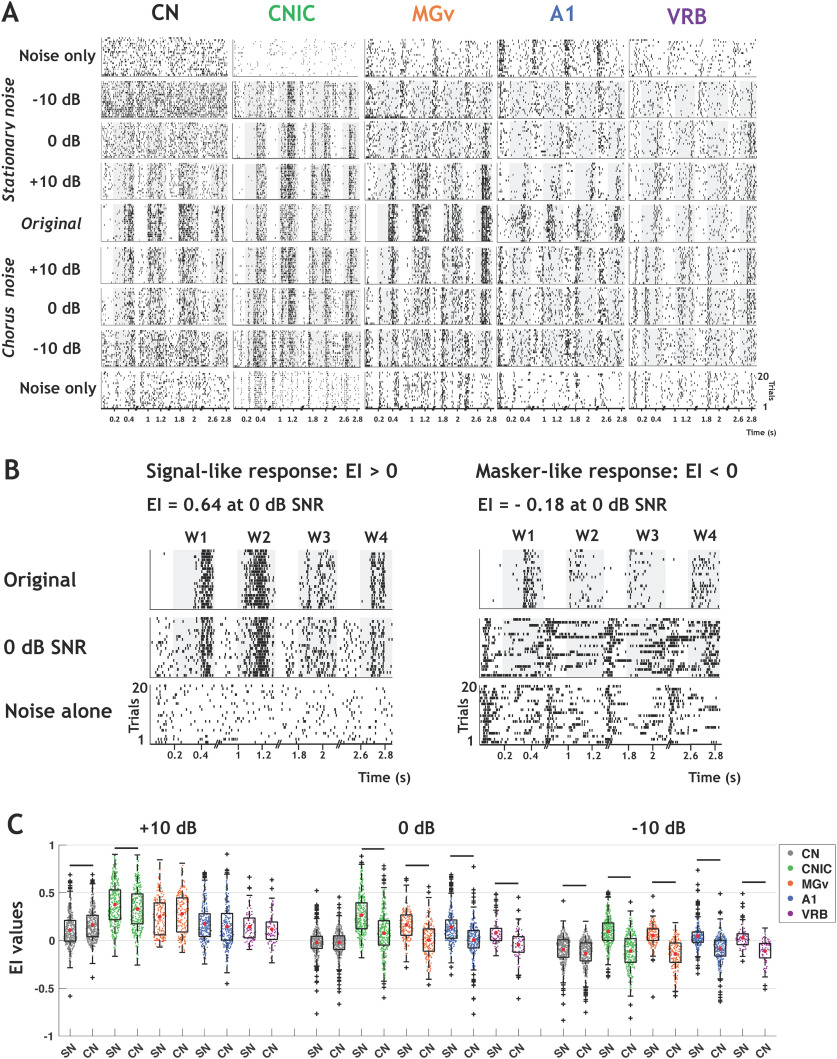
The decrease in EI values is more pronounced in chorus noise than in stationary noise in each auditory structure. ***A***, Raster plots of responses of the four original vocalizations, noisy vocalizations (in both noises), and noise alone recorded in CN, CNIC, MGv, A1, and VRB. The gray part of rasters corresponds to the evoked activity. For each structure, all the rasters correspond to the same recording. ***B***, Rasters showing examples of neuronal responses in stationary noise with values of EI > 0 corresponding to a signal-like response (left, IC recording) and EI < 0 corresponding to a masker-like response (right, A1 recording). Top panels show the responses to the original vocalizations, the middle panels the responses to vocalizations at the 0-dB SNR in stationary noise and the bottom panels the responses to stationary noise alone. ***C***, Box plots showing the EI values for the three SNRs obtained in CN (in black), CNIC (in green), MGv (in orange), A1 (in blue), and VRB (in purple) alternatively in stationary noise (SN) and chorus noise (CN). In each box plot, the red dot represents the mean value. The black lines represent significant differences between the mean values (one-way ANOVAs, *p* < 0.001; with *post hoc* paired *t* tests, *p*^a^_+10 dB,CN_ = 4.03e-18, *p*^b^_+10 dB, CNIC_ = 1.45e-07, *p*^c^_0dB, CNIC_ = 2.47e-45, *p*^d^_0dB, MGv_ = 2.11e-30, *p*^e^_0dB, A1_ = 5.41e-25, *p*^f^_0dB,VRB_ = 6.36e-10, *p*^g^_-10 dB, CN_ = 5.74e-12, *p*^h^_-10 dB, CNIC_ = 1.83e-59, *p*^i^_-10 dB, MGv_ = 1.16e-36, *p*^j^_-10 dB, A1_ = 2.84e-24, *p*^k^_+10 dB, VRB_ = 4.62e-11).

Figure 2*A* shows rasters for recordings collected in the five auditory structures in response to the original (in quiet) and masked vocalizations embedded in stationary (top) and chorus (bottom) noise. In all structures, the neuronal responses evoked by the four whistles progressively vanished as the SNR decreased from +10 to −10 dB. However, one can clearly notice that the recordings obtained in CNIC and MGv still display detectable responses at 0-dB SNR, even down to −10 dB for some vocalizations in CNIC.

To evaluate the neuronal resistance to noise, we quantified the EI (see Materials and Methods; Schneider and Woolley, 2013; Ni et al., 2017) of the 1267 recordings obtained in the five structures. For each recording, the EI compares the PSTH obtained at a given SNR with the PSTHs obtained with the original vocalizations and with the noise alone: the higher the EI value (close to 1), the more the responses are signal-like (Fig. 2*B*, left). Conversely, the lower the EI value (close to −1), the more the responses are masker-like (Fig. 2*B*, right). In both noises, the EI values were higher in the IC and thalamus than in the CN and cortex, except in chorus noise at – 10-dB SNR, which strongly impacted all neuronal responses at each stage (Fig. 2*C*). In addition, the EI values obtained in chorus noise at 0 and −10-dB SNR were significantly lower than those obtained in stationary noise in all structures except in the CN at 0-dB SNR (one-way ANOVAs, *p* < 0.001; with *post hoc* paired *t* tests, *p* < 0.001; Fig. 2*C*).

Thus, in all structures, both noises altered the evoked responses promoting masker-like responses, the chorus noise promoting, on average, a significantly higher proportion of masker-like responses than the stationary noise.

### Robustness to noise is a distributed property in the auditory system

**Figure 3.**
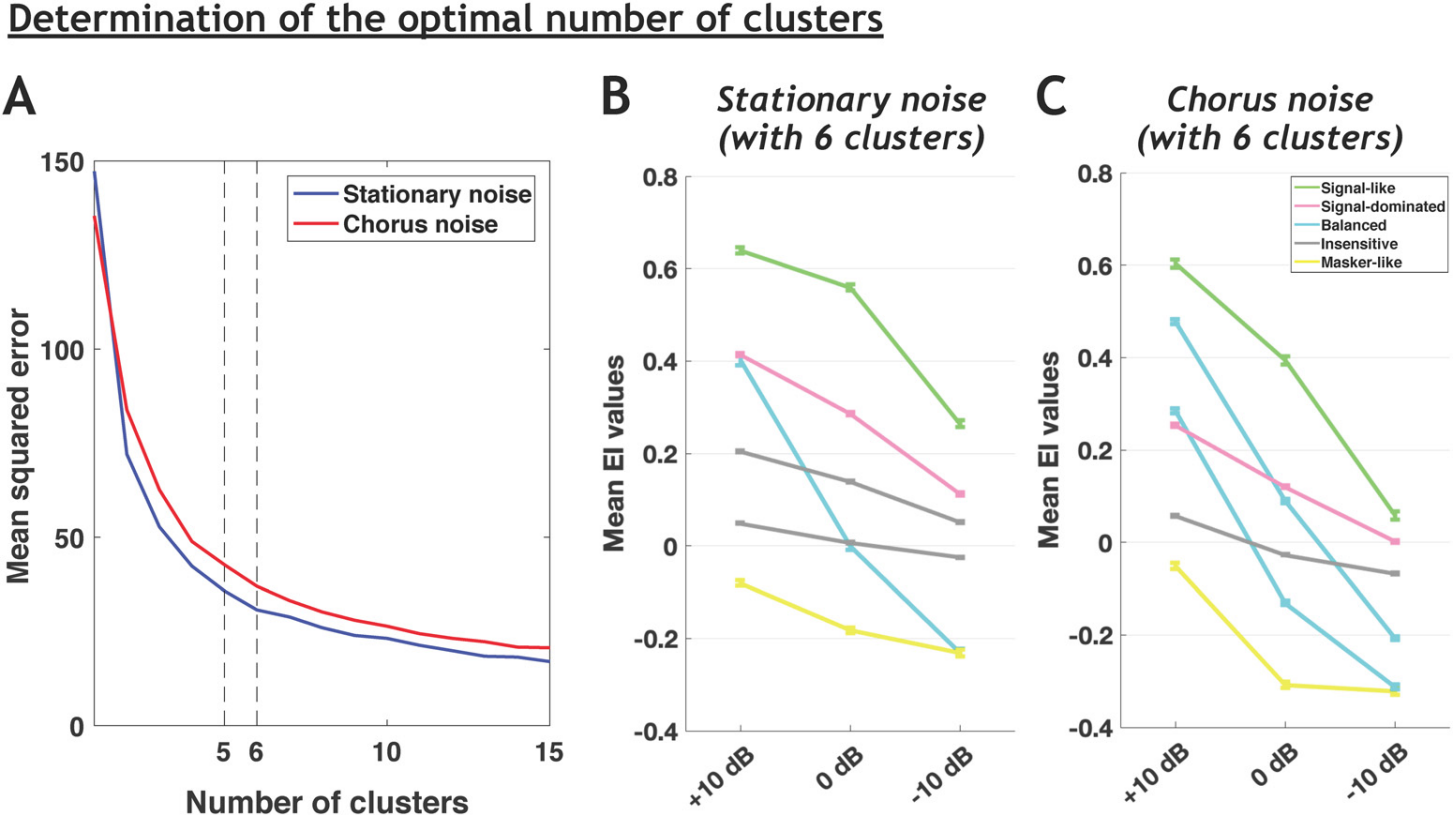
The choice of five clusters is optimal to reveal the different behaviors in both noises. ***A***, Mean square error of EI profile clustering as a function of the number of clusters using the K-means algorithm for the stationary and chorus noise. ***B***, ***C***, Population average EI profile (±SEM) of each cluster when considering six clusters to separate the data in the stationary noise (***B***) and in the chorus noise (***C***). Note that in both noises, two clusters have similar mean EI profile, i.e., the same EI evolution across the three SNRs (the two gray clusters in ***B*** and the two blue clusters in ***C***) leading us to consider only five clusters in the following results (Fig. 4).

We initially aimed at determining whether the four categories of cortical neurons (robust, balanced, insensitive, and brittle) described by Ni et al. (2017) can also be found at each stage of the auditory system. For each neuronal recording, we computed six EI values (three for the stationary noise and three for the chorus noise, relative to the three SNRs). To do the clustering, we used the three EI values of all responses (i.e., the 1267 recordings) separately, in stationary and chorus noise. However, analyzing our whole database with the same clustering method and the same criterion (elbow method) as in Ni et al. (2017) led us to consider either five or six clusters in both noises (Fig. 3), potentially because our recordings came from three subcortical structures in addition to two cortical areas. When six clusters were defined, two of them displayed very similar behaviors with only slight differences in EI values at the three SNRs (see Fig. 3*B*,*C*), which urged us to consider only five clusters and suggests also that a larger number of clusters would have been non-informative as similar behaviors should re-appear. Compared with the four categories of Ni et al. (2017), we added one new category, which represents an attenuated version of their robust category. These neurons cannot be neglected as they represent in fact a large proportion of our database (25% and 18% in the stationary and chorus noise).

**Figure 4.**
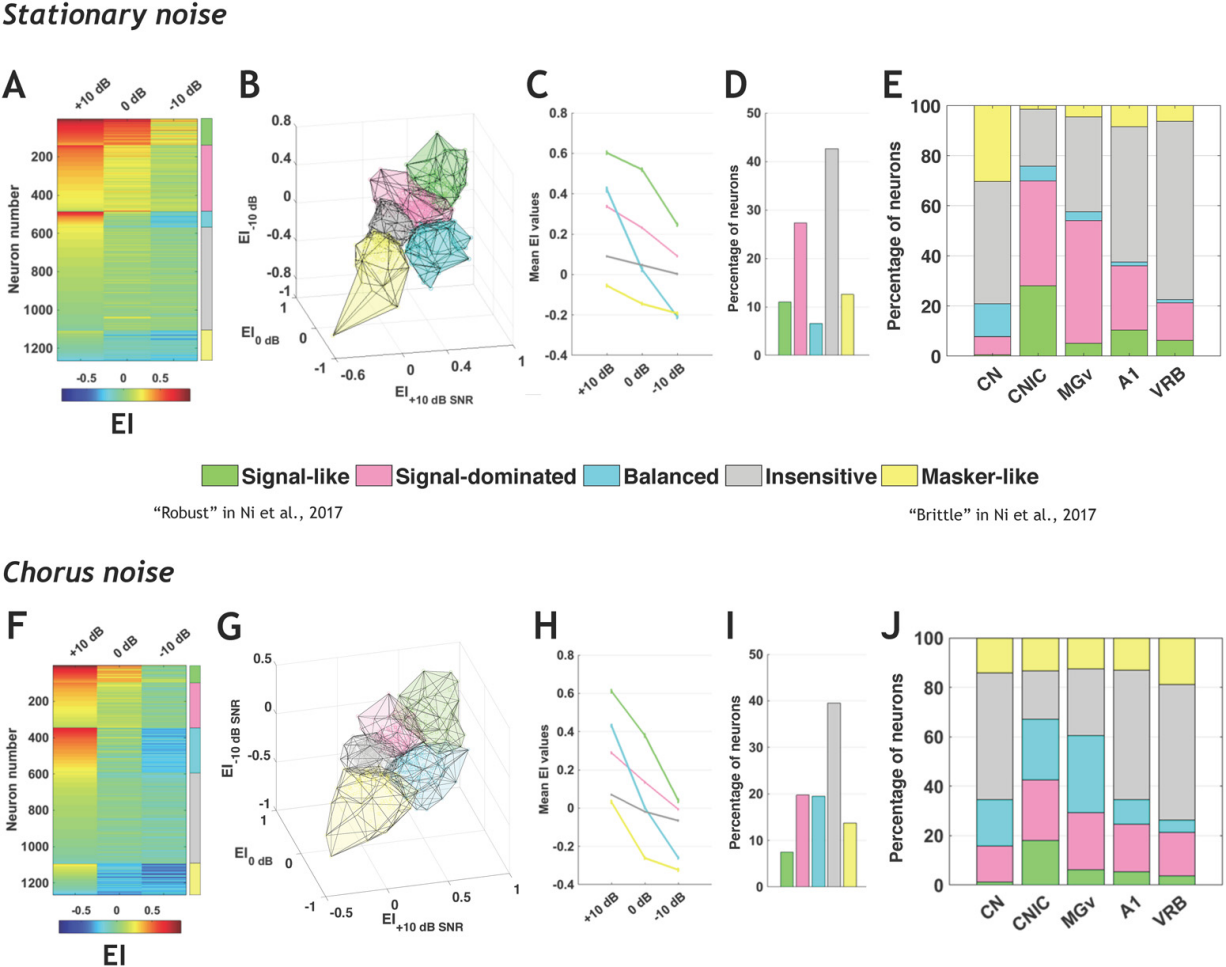
Robustness to noise is a distributed property in the auditory system. ***A***, Each row corresponds to the EI profile of a given neuronal recording obtained in the five auditory structures in stationary noise with a color code from blue to red when progressing from low to high EI values. On the right, five stacked colors delineate the identity for the five categories of responses. The signal-like category is in green, the signal-dominated category in pink, the balanced category in turquoise, the insensitive category in gray and the masker-like category in yellow. The names of the categories used in the study by Ni et al. (2017) are provided for comparison. ***B–E***. 3D representation of the five categories in stationary noise (***B***), mean EI values (±SEM) of the five categories (***C***), relative proportions of each category in stationary noise (***D***), and proportion of each category in the five auditory structures from CN to VRB (***E***). ***F–J***, Same representations as in ***A–E*** for the responses collected in the chorus noise. See Table 1, selection type (b), for referring to the number of selected recordings in each structure.

Figure 4 presents the five categories derived from the whole data set across the three SNRs and the two noises. Figure 4*A*,*F* presents the EI values of all neurons in the three SNRs in a given background noise (with a color code from blue to red when progressing from low to high EI values). The five categories are indicated by a color bar on the right side and are derived from a continuum of EI values. This color code is used for the 3D representations of the five categories in the stationary (Fig. 4*B*) and chorus (Fig. 4*G*) noise. Figure 4*C* shows the mean EI values in stationary noise for these five categories across the three SNRs and the percentage of neurons in each category is displayed in Figure 4*D* ∼10% of the neurons exhibit signal-like responses characterized by mean EI values >0.5 at +10- and 0-dB SNRs. More than 25% display signal-dominated responses characterized by mean EI values >0.2 at +10- and 0-dB SNRs. Approximately 5% of the neuronal responses are balanced and >40% of the total population has a mean EI value around 0 at all SNRs, which corresponds to the insensitive responses. More than 10% of the auditory neurons have negative mean EI values at the three SNRs, which correspond to masker-like responses. Figure 4*H*,*I* shows the mean EI values for these five categories in the chorus noise with, roughly, similar proportions of the five categories as in the stationary noise. However, in the chorus noise there were less signal-like (from 10% to 7.5%) and signal-dominated (from 27% to 20%) responses and more balanced responses (from 6.5% to 19.5%), whereas the proportion of insensitive responses remained similar (42–39.5%). Note also that in the chorus noise, the categories of signal-like and signal-dominated responses showed, on average, lower EI values at the 0- and −10-dB SNR than in stationary noise (compared Fig. 4*C* and *H*). Based on these quantifications performed in the entire auditory system, we found five similar neuronal behaviors in the stationary and chorus noise.

What are the proportions of these categories in each structure? For each auditory structure, the percentage of neurons from each category is presented in stationary noise (Fig. 4*E*) and in chorus noise (Fig. 4*J*). In stationary noise, signal-like and signal-dominated responses were mostly present in the IC and thalamus, while the three other categories of neuronal responses classified as balanced, insensitive, and masker-like were mostly present in the CN and in the two cortical fields. Statistical analyses confirmed that the proportions of the different categories differed in IC and MGv compared with the three other structures (all χ^2^; *p* < 0.001). In chorus noise, there was a large increase in the proportion of balanced responses and a decrease in signal-like responses in all structures, but these latter neurons remained in higher proportions in IC and MGv than in CN and in cortex. Moreover, the masker-like responses were in equivalent proportions in all structures (between 12.5% and 19%). In the CN, there was also an increase in the proportion of signal-dominated responses (from 7% to 14.5%). Statistical analyses confirmed that, in the chorus noise too, the proportions of the different categories differed in the IC and MGv compared with the three other structures (all χ^2^; *p* < 0.001).

Note that including the recordings with no significant TFRP (*n* = 82) led to exactly the same proportions of recordings in the different categories in both noises (Extended Data Fig. 4-1). These neurons, which did not respond to pure tones, displayed no signal-like response in subcortical structures and very rarely in the auditory cortex (see Extended Data Fig. 4-1*D*,*H*). In addition, in an attempt to evaluate the differences between the ventral and dorsal parts of the CN, we compared the EI values for a set of 87 recordings collected at the deepest electrode placements (assuming that they were potentially located in or close to VCN) versus the rest of the CN population considered as collected in DCN (Extended Data Fig. 4-2). The mean EI values did not significantly differ between these two populations at the three SNRs in both noises (Extended Data Fig. 4-2*A*,*B*) and the proportions of the different categories were relatively similar in these two populations. Note also, that in the IC, the recordings potentially obtained from the non-lemniscal divisions displayed (1) more balanced responses than in the lemniscal division in the stationary noise, and (2) more insensitive responses than in the lemniscal division in the chorus noise (Extended Data Fig. 4-3).

10.1523/ENEURO.0043-21.2021.f4-1 Extended Data Figure 4-1 Similar results as in Figure 4 are obtained taking into account the neurons without significant TRFP. ***A–C***, Mean EI values (±SEM) of the five categories across the three SNRs (+10, 0, and –10 dB; ***A***), relative proportions of each category in stationary noise (***B***) and proportion of each category in the five auditory structures from CN to VRB for the recordings with or without significant TFRP (relative to pure tone responses; ***C***). ***D***, Proportion of each category in the five auditory structures from CN to VRB for the recordings without significant TFRP. ***E–H***, Same representations as in ***A–D*** for the chorus noise. See Table 1, selection type (a), for referring to the number of selected recordings in each structure. Download Figure 4-1, TIF file.

10.1523/ENEURO.0043-21.2021.f4-2 Extended Data Figure 4-2 Attempt to separate the ventral and dorsal parts of the CN based on the depth of the recordings. ***A***, ***B***, Box plots showing the EI values for the three SNRs obtained in the ventral (VCN, in black) and dorsal (DCN, in grey) parts of the CN in stationary noise (***A***) and chorus noise (***B***). In each box plot, the red dot represents the mean value. There was no significant difference between the EI values obtained in VCN and DCN for the three SNRs and in both noises (one-way ANOVAs, *p* > 0.05; with *post hoc* paired t tests, *p* > 0.05). ***C***, Proportion of each category in the ventral part of the CN (VCN, *n* = 87 recordings) and its dorsal part (DCN, *n* = 302 recordings) obtained in stationary (SN) and chorus (CN) noise. Download Figure 4-2, TIF file.

10.1523/ENEURO.0043-21.2021.f4-3 Extended Data Figure 4-3 Lemniscal and non-lemniscal parts of the IC. Proportion of each category in the lemniscal part of IC (CNIC, *n* = 339 recordings) and its non-lemniscal parts (dorsal and external cortices of the IC, DCIC-ECIC, *n* = 73 recordings) obtained in stationary and chorus noise. See Table 1, selection type (b), for referring to the number of selected recordings. Download Figure 4-3, TIF file.

To sum up, in both noises, the neurons with a high-fidelity representation of the signal were mostly present at two subcortical levels, in the IC and thalamus. The insensitive responses showing no preference either for the signal or the noise were found in majority in the CN and in the auditory cortex. The balanced responses represented a small fraction of neurons in stationary noise but were more numerous in the chorus noise, especially in IC and MGv. Interestingly, in both types of noise, the proportion of these balanced responses decreased as one ascends in the auditory system suggesting a decrease in sensitivity to SNR at the cortical level. Finally, the neurons with a high-fidelity representation of the noise were mostly localized in the CN in the stationary noise but were in an equivalent proportion in all structures in the chorus noise (between 12.5% and 19%).

### The noise-type sensitivity is found at each stage of the auditory system

In the auditory cortex of awake marmoset, Ni et al. (2017) have pointed out that the neuronal behavior in noise can be context dependent: the behavior of a given neuron in a particular noise does not predict its behavior in another noise. Is this a property characterizing cortical neurons, or is it a general property that exists at all levels of the auditory system?

**Figure 5.**
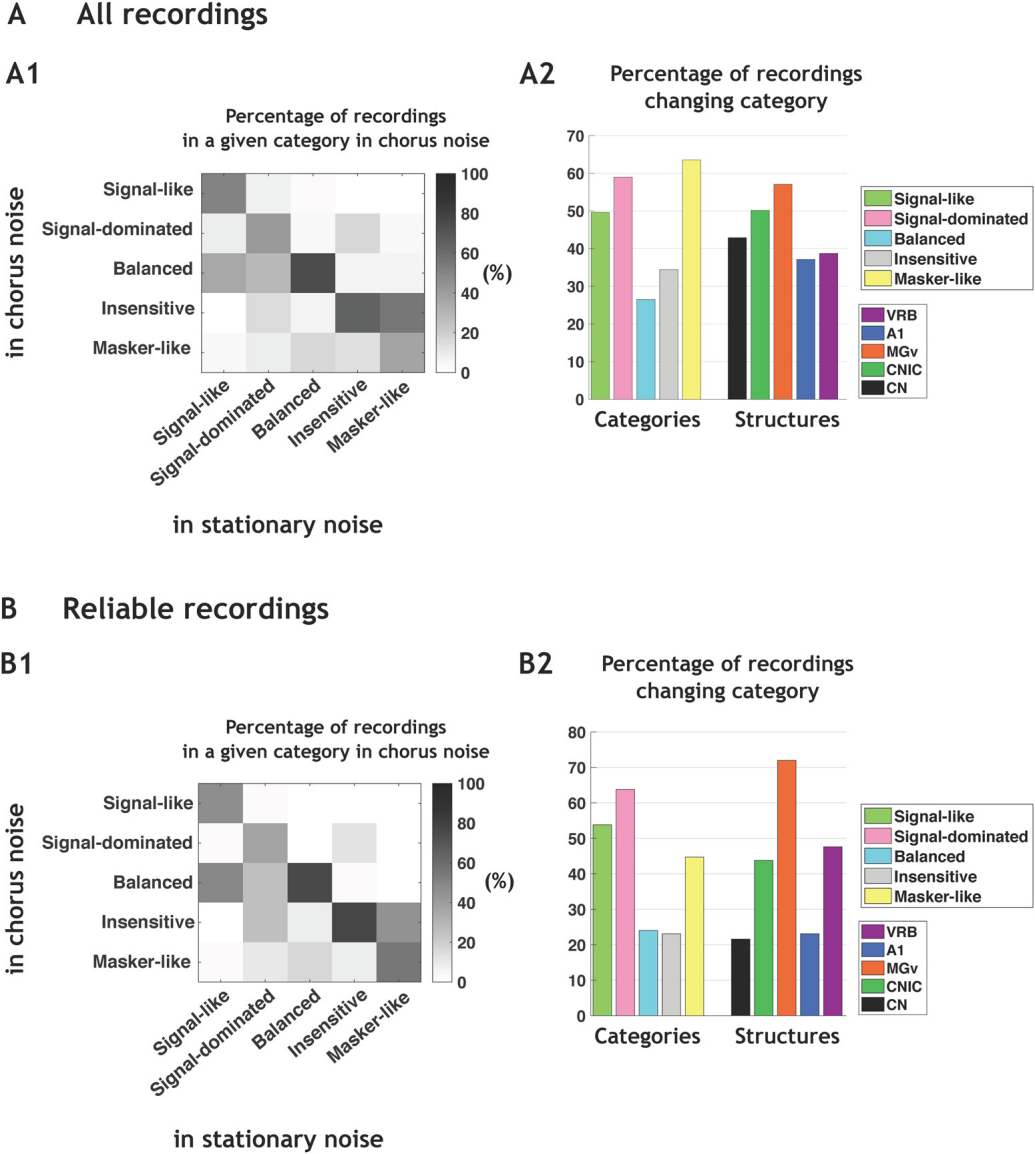
The noise-type sensitivity is found at each stage of the auditory system. ***A1***, Group-switching matrix representing the percentage of recordings in a given category in chorus noise depending on the category originally assigned in the stationary noise. The abscissas indicate the category identity in the stationary noise, and the ordinates represent the category identity in the chorus noise. For example, signal-like responses in stationary noise are also 50% signal-like in chorus noise but 10% are reclassified as signal-dominated, 35% balanced, 1.5% insensitive, and 3.5% masker-like. Note that, in stationary noise, the number of recordings in each category were 139, 346, 83, 540, and 159 in signal-like, signal-dominated, balanced, insensitive, and masker-like category, respectively. ***A2***, Mean percentages of recordings changing category from the stationary noise to the chorus noise, first in each category and second in each structure [VRB, (in purple), A1 (in blue), MGv (in orange), CNIC (in green), and CN (in black)]. ***B1***, Group-switching matrix representing the percentages of recordings changing category from the stationary noise to the chorus noise based only on recordings considered as reliable with the bootstrap procedure in the two types of noise (with a confidence interval ⩾95%). ***B2***, Mean percentages of recordings changing category from the stationary noise to the chorus noise, first in each category and second in each structure [VRB, (in purple), A1 (in blue), MGv (in orange), CNIC (in green), and CN (in black)].

In each auditory structure, the neuronal behaviors were partly, but not completely, preserved in the two noises. In Figure 5*A1*, the group-switching matrix represents the percentage of neurons in a given cluster in chorus noise depending on the cluster originally assigned in the stationary noise. The preservation of the same neuronal behavior in both noises is indicated by the percentages in the diagonal line. Approximately 50% of the signal-like and 40% of the signal-dominated neuronal responses in the stationary noise remained so in the chorus noise. Most of the balanced (73.5%) and insensitive neuronal responses (65.5%) in the stationary noise remained also in the same category in the chorus noise. Only 36.5% of the masker-like neuronal responses remained so in the chorus noise. Figure 5*A2* indicates that the signal-like, signal-dominated and masker-like neuronal responses were the three categories with the highest percentages of category changes (χ^2^ *p* < 0.001). In the different structures, the percentage of category changes was between 37% and 57% without significance difference between structures.

A bootstrap procedure was used to estimate the percentage of category changes, which can occur simply because of response variability (see Materials and Methods). We suspected that, independently of the change in noise type, a recording can potentially change category because of its response variability and/or because it was located at the frontier between two clusters. Briefly, for each recording, and from the pool of 20 trials obtained for each stimulus, we resampled 20 trials (allowing repetitions), recomputed the EI and reallocated each resampled recording in the closest category. This entire procedure was performed 100 times for each recording. In all the following results, we only considered the recordings which remained in the same category 95 times or more (out of the 100 bootstraps) in both noises, that is, recordings that displayed a very high reliability of their responses and were assigned to a particular category with a 95% confidence or more.

Figure 5*B1* shows, for this population of 342 recordings, that a non-negligible fraction (36–75%) of the neurons assigned to a given category remained in the same category when shifting from the stationary to the chorus noise. When analyzing the percentage of neurons changing categories, we found a similar pattern as the whole population of 1267 neurons, i.e., the largest proportions of neurons switching category from the stationary to the chorus noise were in the signal-like, signal-dominated and masker-like categories (χ^2^ *p* < 0.05; Fig. 5*B2*). Analyzing the percentage of neurons changing clusters according to the structure revealed that the lowest percentages of category changes were in the CN and in A1 (21% and 22%) and the highest proportion in MGv (72%, χ^2^ *p* < 0.05).

Note that when computing the MI based on all neurons (Extended Data Fig. 5-1*A*) or only reliable neurons (Extended Data Fig. 5-1*B*), we obtained low MI values (0.53 bits for all neurons and 0.7 bits for reliable neurons) confirming that a large proportion of neurons change category between noises.

10.1523/ENEURO.0043-21.2021.f5-1 Extended Data Figure 5-1 Confusion matrices obtained for all and reliable recordings. ***A***, Confusion matrix relative to Figure 5*A1* representing the number of recordings in a given category in chorus noise depending on the category originally assigned in the stationary noise. ***B***, Same as in ***A*** with only recordings considered as reliable with the bootstrap procedure in the two types of noise (with a confidence interval ⩾95%). This figure is relative to the Figure 5*B1*. Note that the MI (bits) values were low in ***A***, ***B*** corroborating the fact that a large proportion of recordings assigned to a given category in stationary noise change category in chorus noise. Download Figure 5-1, TIF file.

Thus, even when using a bootstrap procedure with a severe selection criterion, a non-negligible fraction of recordings changes categories from one background noise to another and this noise-type sensitivity is found at each stage of the auditory system.

### The neuronal behaviors in stationary and chorus noise are predictable based on response parameters obtained in quiet

A fundamental question is whether the assignment of a given recording to a particular category can be predicted from the response characteristics obtained by presenting pure tones and/or the original vocalizations in quiet and/or the maskers alone. To address this question, we firstly focused on the neurons reliably categorized with the bootstrap procedure in both noises (with a confidence interval > 95%, *n* = 342).

To determine whether the assignment of a recording to a particular category can be predicted based on response characteristics, we trained an artificial classifier (LDA) with all combinations of descriptor types.

Three descriptors were extracted from the responses to pure tones (TFRP): the BF firing rate, the bandwidth of tuning and the response duration. From the responses to the signal alone (original vocalizations) and the maskers alone, two descriptors were extracted: the firing rate and the temporal reliability (CorrCoef index; see Materials and Methods). Three other masker descriptors were used in both noises in an attempt to quantify the firing rate short-term adaptation to the masker (see Materials and Methods). Finally, we included two descriptors corresponding to the ratios between the responses to the signal and to the masker (see Materials and Methods).

**Figure 6.**
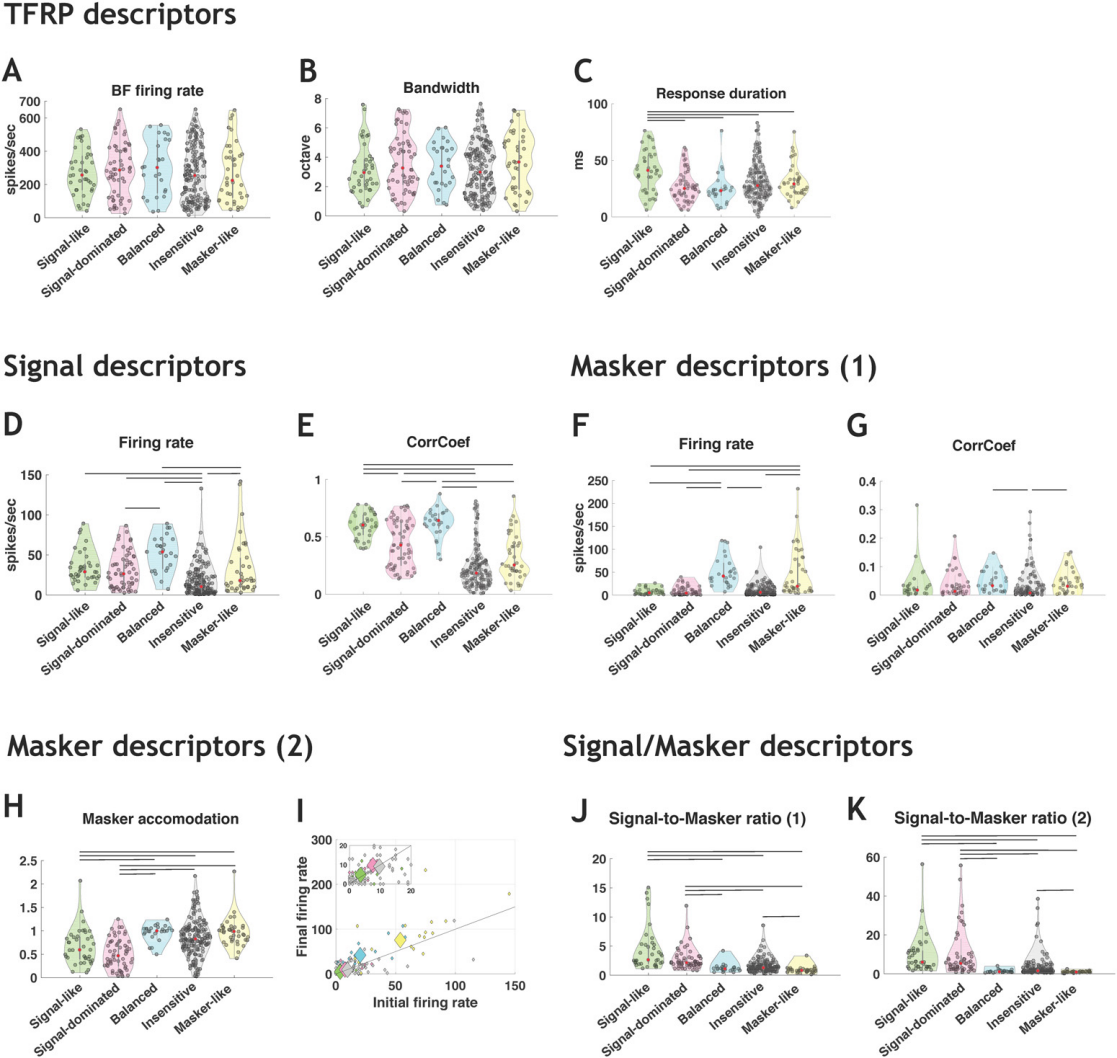
Descriptors of the categories in stationary noise used by the classifiers. ***A–C***, Three TFRP parameters were chosen as descriptors: the BF firing rate, the bandwidth and the response duration. ***D***, ***E***, Two signal descriptors were selected corresponding to the firing rate and the CorrCoef values obtained in original conditions. ***F***, ***G***, Two main masker descriptors were presented corresponding to the firing rate and the CorrCoef values obtained in stationary noise alone. ***H***, ***I***, The three other masker descriptors are: (***H***) the ratio between the masker firing rate taken at the time the signal should have occurred and the initial masker firing rate during the first 200 ms of the masker and the number of action potentials emitted during the first (initial; ***I***) and last (final, ***I***) 50 ms of the masker alone over a 564-ms period. ***J***, ***K***, Two descriptors of the signal-to-masker ratio are presented and taken into account the firing rate of responses to the signal and to the masker; the two differ only on which part of the response to the masker is taken into account (see Materials and Methods). For each violin plot, the red dot represents the median value and the black lines represent significant differences between the median values (Kruskal–Wallis test, *p* < 0.05).

In stationary noise, for the descriptors extracted from the TFRP (Fig. 6*A–C*), the distributions of BF firing rate and bandwidth of tuning did not point out significant differences across the five categories (Fig. 6*A*,*B*), but the signal-like category showed significantly longer response duration than the other categories (Kruskal–Wallis test, *p* < 0.05; Fig. 6*C*). For the descriptors extracted from the signal responses (Fig. 6*D*,*E*), the firing rate was significantly lower for the insensitive responses compared with all other response categories (Kruskal–Wallis test, *p* < 0.05; Fig. 6*D*) and the signal-like and balanced responses had significantly higher CorrCoef values compared with three other categories (Kruskal–Wallis test, *p* < 0.05; Fig. 6*E*). For the descriptors extracted from the responses to the masker (Fig. 6*F–I*), the signal-like, signal-dominated and insensitive neuronal responses showed lower firing rate compared with the balanced and masker-like responses (Kruskal–Wallis test, *p* < 0.05; Fig. 6*F*), whereas the CorrCoef values did not differ across categories except for the insensitive neuronal responses that showed the lowest CorrCoef values (Kruskal–Wallis test, *p* < 0.05; Fig. 6*G*). The masker accommodation (Fig. 6*H*,*I*) was significantly lower for the signal-like and signal-dominated categories than the other categories, indicating that these two categories adapted more to the masker (their firing rate decreased during presentation of the masker alone). For the two descriptors combining the firing rate to the signal and to the masker (Fig. 6*J*,*K*), the values were higher for the signal-like and signal-dominated categories than for the other categories (Fig. 6*J*,*K*) indicating that these two categories displayed marked preference for the signal over the masker (Kruskal–Wallis test, *p* < 0.05; Fig. 6*G*).

**Figure 7.**
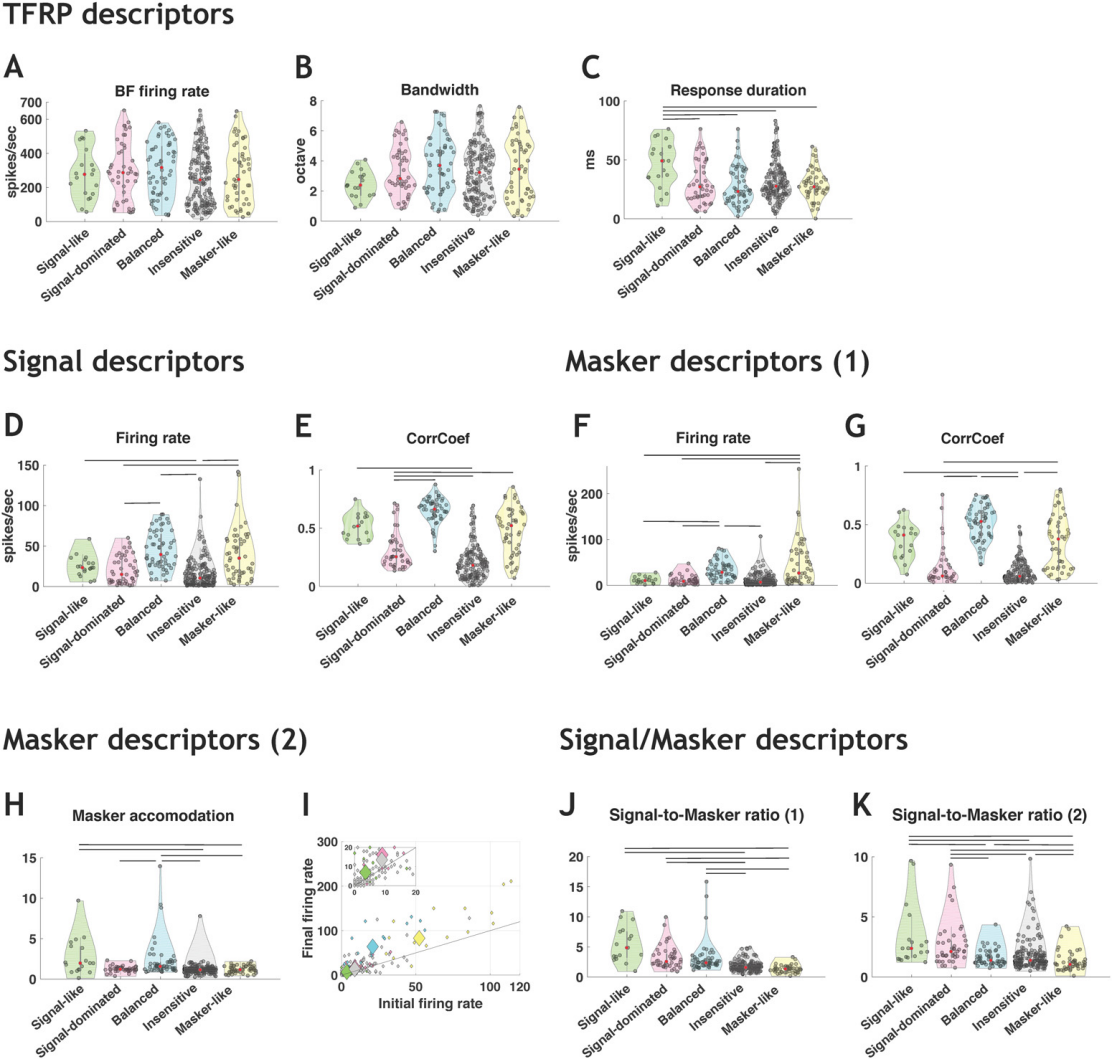
Descriptors of the categories in chorus noise used by the classifiers. ***A–C***, Three TFRP parameters were chosen as descriptors: the BF firing rate, the bandwidth and the response duration. ***D***, ***E***, Two signal descriptors were selected corresponding to the firing rate and the CorrCoef values obtained in original conditions. ***F***, ***G***, Two main masker descriptors were presented corresponding to the firing rate and the CorrCoef values obtained in chorus noise alone. ***H***, ***I***, The three other masker descriptors are: (***H***) the ratio between the masker firing rate taken at the time the signal should have occurred and the initial masker firing rate during the first 200 ms of the masker and the number of action potentials emitted during the first (initial; ***I***) and last (final; ***I***) 50 ms of the masker alone over a 564-ms period. ***J***, ***K***, Two descriptors of the signal-to-masker ratio are presented and taken into account the firing rate of responses to the signal and to the masker; the two differ only on which part of the response to the masker is taken into account (see Materials and Methods). For each violin plot, the red dot represents the median value and the black lines represent significant differences between the median values (Kruskal–Wallis test, *p* < 0.05).

In general, similar results were obtained in the chorus noise for TFRP and signal descriptors (Fig. 7*A–E*). However, more differences emerged across categories for the masker descriptors as the CorrCoef index (Fig. 7*G*). The differences observed across categories were relatively comparable to the differences observed in the CorrCoef values obtained for the signal (Fig. 7*E*).

Altogether, these analyses pointed out that there were little or no between-category differences based on the TFRP descriptors and that the descriptors combining responses to the signal and to the maskers provide information allowing a correct separation between the signal-like and signal-dominated categories versus the others.

**Figure 8.**
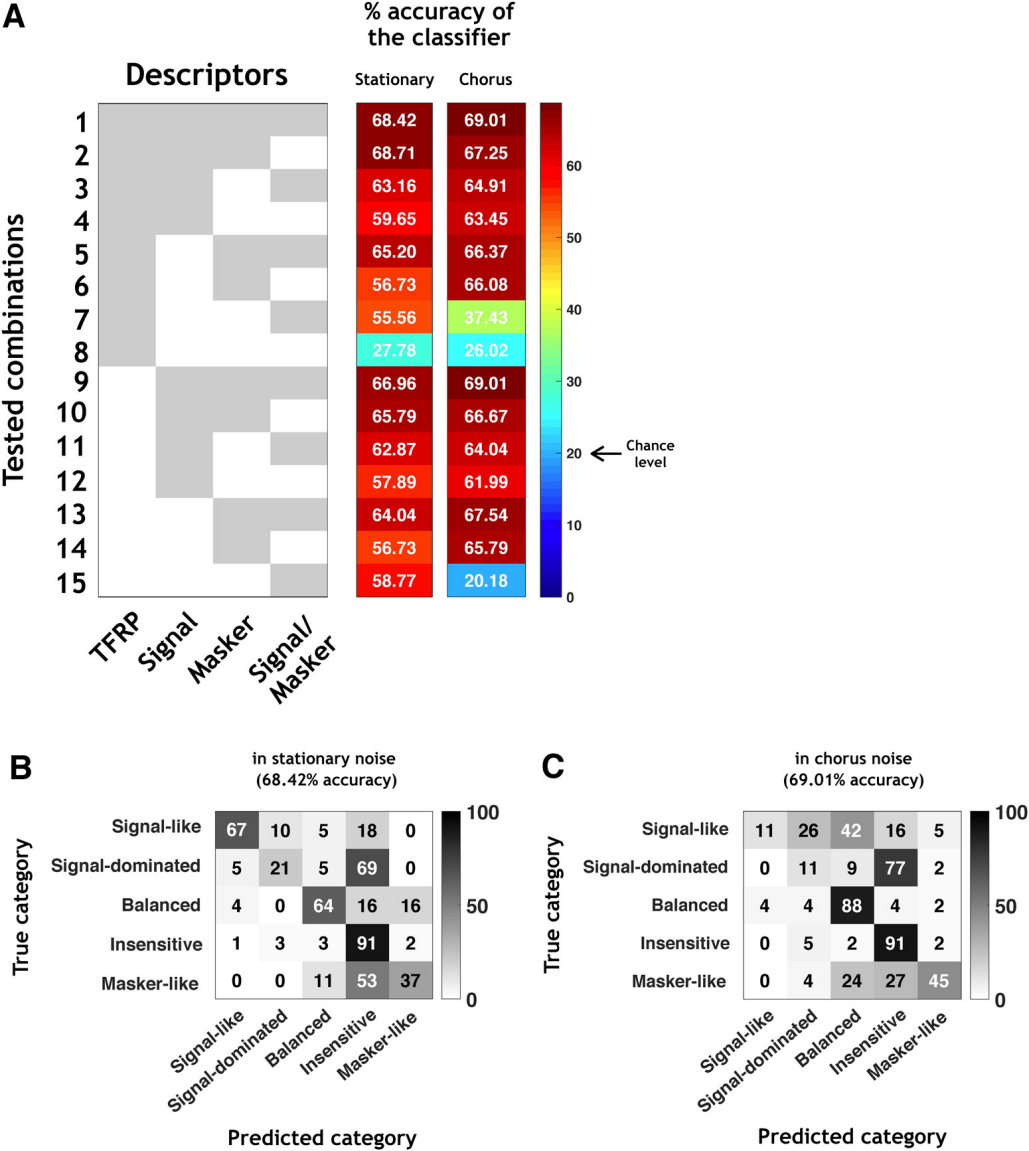
The neuronal behaviors in stationary and chorus noise are predictable based on response parameters obtained in quiet. ***A***, All tested combinations (1–15) based on four types of descriptors (TFRP, signal, masker, and signal/masker) of the categories in stationary noise and their respective percentages of accuracy of the classifier. The gray part means that the descriptor is included in the classifier and the white part means that the descriptor is excluded from the classifier. ***B***, ***C***, Example of the confusion matrix obtained with all descriptors (combination 1) in stationary (***B***) and chorus (***C***) noise. Each row corresponds to a true category and each column corresponds to a predicted category. The numbers in the confusion matrix correspond to the percentage of recordings of a given true category which have been predicted to belong to a given predicted category.

When the classifier was trained with all the descriptors in stationary noise (combination 1 in Fig. 8*A*, left column), the accuracy of the classifier reached 68.42%, which is more than three times the chance level (20% as there were five categories). Figure 8*B* presents the confusion matrix obtained with this classifier in stationary noise and revealed that the percentage of correct classification depends on the category. Indeed, the signal-like, the balanced and the insensitive neuronal responses were well predicted (67%, 64%, and 91%, respectively) whereas only 21% and 37% of the signal-dominated and masker-like neuronal responses were correctly predicted.

Next, for isolating which type of descriptors allowed the higher predictability, we used 14 different classifiers corresponding to the 14 possible combinations from the four types of descriptors (Fig. 8*A*, combinations 2–15). Several important results emerged from these analyses. First, the TFRP descriptors alone (Fig. 8*A*, line 8) led to the lowest accuracy, 27.78%, which is close from the chance level indicating that the responses to pure tones are insufficient to predict the behavior of a given neuron in noise. Second, the descriptors extracted either from the responses to the signal alone (Fig. 8*A*, line 12), or from the responses to the masker alone (Fig. 8*A*, line 14), or from the signal-to-masker alone (Fig. 8*A*, line 15) led to an accuracy between 56.73% and 58.77%, which was only 10% less than the best performance when combining all the descriptors. Finally, the descriptors extracted from the responses to the signal, to the masker and to the signal-to-masker ratio (Fig. 8*A*, line 9), generated an accuracy of 66.96%, which is close from the global level reached with all the descriptors. Therefore, the classifier reached a relatively good performance in stationary noise (66.96%) by combining the three descriptors extracted from the responses to the signal alone and to the masker alone, the TFRP descriptors only slightly increasing the performance of the classifier (around 3%).

Globally, the results were similar in the chorus noise: based on all the descriptors, the accuracy of the classifier was 69%, it dropped to 26% with the TFRP descriptors alone, and between 62% and 66% with the signal descriptors alone or masker descriptors alone (Fig. 8*A*, left column). The only difference was that the accuracy of the classifier with only the signal/masker descriptors was at the chance level (20.18%) probably because the firing rate to the masker is very close to the firing rate to the signal, suggesting that with a masker composed by a mixture of different type of signals (as the chorus noise), the masker firing rate does not significantly increase the performance of the classifier. Figure 8*C* illustrates the confusion matrix obtained by combining the four types of descriptors in chorus noise, which led to the maximum accuracy of the classifier (69.01%).

**Figure 9.**
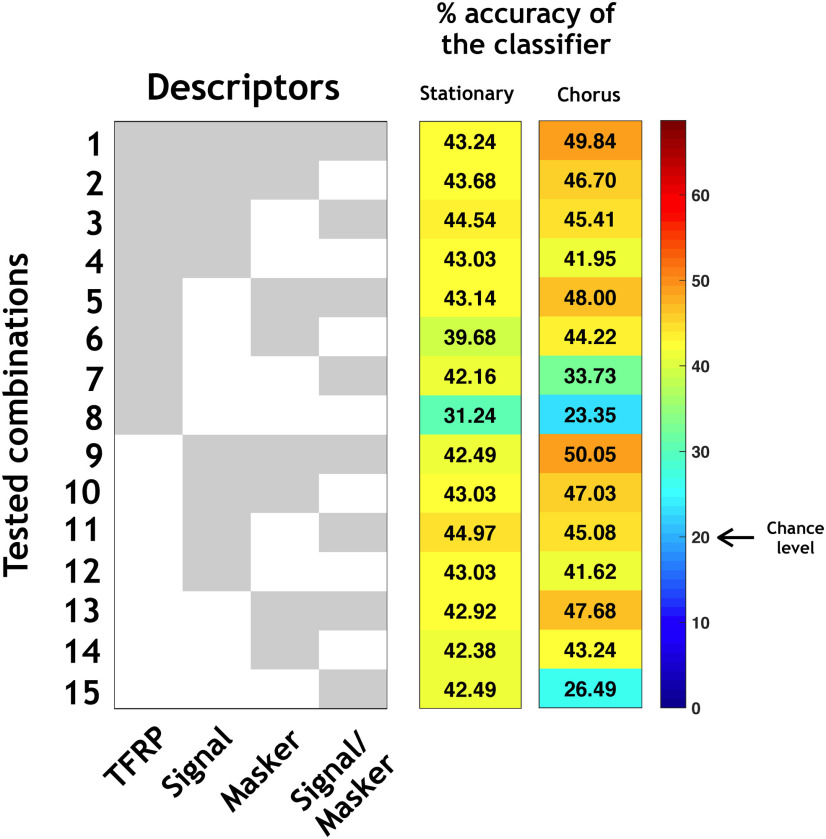
Generalization of the classification. In this figure, the classifiers were trained with the reliable neurons and tested on the rest of the population. All tested combinations (1–15) based on four types of descriptors (TFRP, signal, masker, and signal/masker) of the categories in stationary and chorus noise and their respective percentages of accuracy of the classifier. The gray part means that the descriptor is included in the classifier and the white part means that the descriptor is excluded from the classifier.

To verify that the descriptors are relevant predictors on the whole set of recordings, we trained the classifiers on the reliable neurons and tested them on the rest of the population (Fig. 9). The accuracy of the classification was lower in both noises (43.24% and 49.84% in the stationary and chorus noise, respectively; Fig. 9, combination 1) certainly because among our recordings, some did not display reliable enough responses. However, these values of accuracy were still more than twice the chance level. Again, the descriptors based on the TFRP provided an accuracy close to the chance level (31.24% and 23.35% in the stationary and chorus noise, respectively; Fig. 9, line 8) and the descriptors based on the response to the signal alone (Fig. 9, line 12) and response to the masker alone (Fig. 9, line 14) generated an accuracy that was close from those obtained with the whole set of descriptors (43.03% and 42.38% in stationary noise; 41.62% and 43.24% in chorus noise).

Altogether, our results pointed out that very few neuronal parameters (as the firing rate and the temporal reliability) to the signal alone and to the noise alone are sufficient to predict, up to a maximum of around 70%, the neuronal behaviors in noise.

## Discussion

Here, we demonstrate that the robustness to noise of neuronal responses relies on a distributed network along the auditory system. Signal-like and signal-dominated responses were detected at each level of the auditory system but were in higher proportions at the collicular and thalamic levels. In terms of proportions, the highest fidelity representation of the signal or the noise was found at the subcortical level whereas at the cortical level, the majority of neuronal responses showed no preference for the signal or the noise suggesting that cortical neurons are less sensitive to the spectro-temporal details of the noisy vocalizations. Our results also indicate that neurons sensitive to the type of noise are present at each stage of the auditory system. Finally, a few neuronal parameters extracted from both the responses to the signal alone and to the noise alone convey enough information to predict the neuronal behavior in noise up to a maximum of 70%.

### Limitations of the study

Based on all EI values, the five categories rather form a continuum with no strict boundaries between them, which inevitably led us to « impose » the categories. Nonetheless, using a severe criterion of the bootstrap testing (a confidence interval >95%), we found reliable neurons in each category, in each structure and in both noises. In addition, for a given recording, prediction about its assignment to a particular category reached about a maximum of 70% based on a few descriptors of neuronal responses. All these results suggest that these five behaviors do exist in the whole auditory system. The behaviors of cortical neurons found here in anesthetized animals were the same as previously described in awake marmosets (Ni et al., 2017). Therefore, the cortical representation of noisy signals by different neuronal categories characterized either by the preference of the signal, the masker, a sensitivity to SNR or an absence of these three acoustic features, seems independent of the fact that the animal is awake or anesthetized.

One can suspect that the higher proportion of signal-like responses in the CNIC and MGv compared with auditory cortex, results from the fact that our data were collected in anesthetized animals. According to this view, if the recordings have been collected in awake, behaving animals, the results would be reversed, with a higher proportion of cortical neurons exhibiting robust responses to acoustic degradations. Confrontation of several results suggests that this simple explanation might not being correct. First, Lohse et al. (2020) have recently demonstrated that collicular neurons of awake mice displayed the same gain control adaptation to the stimulus statistics than in anesthetized mice.

Second, auditory cortex responses collected in behaving ferrets were found to be sufficiently robust to preserve vowel identity across a large range of acoustic transformations, such as changes in fundamental frequency, sound location or level (Town et al., 2018). However, earlier studies from the same laboratory performed in anesthetized conditions (Bizley et al., 2009; Walker et al., 2011) have reached similar conclusions for vowels varying in fundamental frequency and virtual acoustic location, indicating that the general principles allowing neuronal discrimination are observable across anesthetized and behavioral states. Furthermore, at the subcortical level, it seems that there is not a large difference between the phase-locking properties in anesthetized and awake animals. In fact, in awake animals, the subcortical neurons, especially collicular ones (Ter-Mikaelian et al., 2007), will still be far better than cortical ones to follow the 4- to 20-Hz temporal cues contained in the four vocalizations, which are crucial cues for responding to these signals in noisy conditions (see Souffi et al., 2020; their Fig. 12). Thus, the differences observed here between cortical and subcortical structures in detecting and responding to communication sounds in noisy conditions should remain the same in awake preparations.

Using the same methods to classify and determine the optimal number of clusters as Ni et al. (2017), we opted for five categories rather than four, which is one main difference with their study. Our additional category corresponds to the signal-dominated responses and stands as an intermediate category between the signal-like and the insensitive responses. These responses might represent a too small fraction in their cortical data to emerge as a category, but the inclusion of three subcortical structures and a secondary cortical area might favored their emergence in larger proportions (25% and 18% in the stationary and chorus noise, respectively). We can also wonder whether choosing seven, eight, or nine clusters, would have highlighted other neuronal behaviors. Part of the answer is provided by Figure 3*B*,*C*, which show that with six clusters, similar behaviors re-appear suggesting that a larger number of categories would have been non-informative. However, it is possible that more specific behaviors might have been missed as we collected multiunit recordings composed of two to six shapes of action potentials. This is potentially the case at the cortical level where a large number of cell types have been described (Ascoli et al., 2008; DeFelipe et al., 2013) and also in the CN (Cant and Benson, 2003; Kuenzel, 2019). In fact, in the CN, multiunit recordings might have masked the distinct temporal response profiles corresponding to different morphologic cell types. For example, the pause/build-up cells have been associated with the fusiform cells in the dorsal division of CN (Rhode et al., 1983; Smith and Rhode, 1985), whereas primary-like, onset and phase-locked patterns have been associated with the VCN globular bushy cells (Smith and Rhode, 1985). Because of our surgical approach, our recordings mainly (but not exclusively) come from the DCN.

Obviously, it is also important to assess whether our results can be generalized to other types of communication sounds and to other types of masking noises. In fact, similar results were found in the initial study by Ni et al. (2017) with five other different vocalizations and two other masking noises which suggests that with our experimental conditions, the results should not significantly change, at least in A1, with other types of communication sounds and other masking noises.

### Robustness to noise in the auditory system: a localized versus distributed property?

In the A1 of awake marmosets, Ni et al. (2017) found ∼20–30% of robust responses (depending on the vocalization), called here signal-like responses. In our cortical data, when pooling together the signal-like and signal-dominated responses, we obtained about the same proportions as in the marmoset A1 (33%). In the bird auditory system, Schneider and Woolley (2013) described the emergence of noise-invariant responses for a subset of cells (the broad spike cells) of a secondary auditory area (Caudomedial Nidopallium, area NCM), whereas upstream neurons (IC and A1 neurons in their study) represent vocalizations with dense and background-corrupted responses. They suggest that a sparse coding scheme operating within NCM allows the emergence of this noise-invariant representation. In our study (and in the mammalian A1 in general), a sparse representation already exists as early as A1 (see the rasters in Fig. 2*A*; see also Hromádka et al., 2008) allowing signal-like and signal-dominated responses to be present in about the same proportions in A1 and in the secondary area VRB.

Noise-invariant representations were also reported in A1 of anesthetized ferrets (Rabinowitz et al., 2013). This study suggested a progressive emergence of noise-invariant responses from the auditory nerve to IC and to A1, and proposed the adaptation to the noise statistics as a key mechanism to account for the noise-invariant representation in A1. However, Lohse et al. (2020) have recently challenged this result by showing, in anesthetized animals too, (1) that collicular, thalamic and cortical neurons display the same adaptation to noise statistics; and (2) importantly, that silencing the auditory cortex did not affect the capacity of IC and MGv neurons to adapt to noise statistics.

In fact, Las et al. (2005) reported that A1, MGB, and IC neurons can detect low-intensity target tones in a louder fluctuating masking noise and display the so-called “phase-locking suppression,” that is the interruption of phase-locking to the temporal envelope of background noise. This last result indicates that both IC and MGB neurons have the same ability as cortical neurons to detect low-intensity target sounds in louder background noises (even at −15- or −35-dB SNR). Thus, the robustness of some of our subcortical neurons may stem from this ability to detect the vocalizations even at SNRs as low as the −10-dB SNR.

Based on the proportion of signal-like and signal-dominated responses, it seems that the robustness to noise peaks in CNIC, with the MGv neurons being at the intermediate level between IC and A1 (Fig. 4*E*,*J*). In fact, our results point out an abrupt change from a prominent noise-sensitivity in CN to a prominent noise-robustness in IC, which means that this robustness is generated by neural computation taking place in the central auditory system. Whether this is an intrinsic property emerging *de novo* in the IC or whether this property emerges as a consequence of the multiple inputs converging on IC cells (Malmierca and Ryugo, 2011) remains to be determined. Several studies have clearly demonstrated that IC neurons adapt to the stimulus statistics. First, adaptations of IC neurons to the average stimulus intensity, stimulus variance and bimodality that has already been described with a temporal decay of ∼160 ms at 75 dB (Dean et al., 2005, 2008). Second, adaptation to the noise statistics shifted the temporal modulation function (TMF) of IC neurons to slower modulations, sometimes transforming bandpass TMF to low pass TMF in ∼200 ms of noise presentation (Lesica and Grothe, 2008). In addition, recent studies have shown that a tone-in-noise discrimination task influences neuronal activity as early as the IC (Slee and David, 2015; Shaheen et al., 2021), suggesting that subcortical structures may participate to complex auditory tasks and should not be considered as passive relays.

A particularly interesting result is that, in both types of noise, the proportion of neurons classified as balanced (i.e., showing strong SNR dependence) decreased progressively as one ascends in the auditory system, which is in line with the idea that cortical neurons are less sensitive to SNR than subcortical ones.

### Noise-type sensitivity and noise representation in the auditory system

Ni et al. (2017) found about two-thirds of A1 neurons switching category from one background noise to another, suggesting that the majority of cortical neurons have a behavior specific to the type of noise. We preferred to call this phenomenon noise-type sensitivity rather context-dependence (proposed by Ni et al. 2017) because the latter refers to situations where the same stimulus is presented in different contexts; whereas inserting signal stimuli in two types of noise generated different auditory streams.

Here, we confirmed that these neurons exist at the level of the A1 and extended this result to the subcortical structures. Therefore, different types of noise streams activate different subpopulations of neurons at each stage of the auditory system for constructing invariant representations of communication sounds in noise. In addition, based on a restrictive population of neurons that have a high response reliability as they remained in the same category with a bootstrap procedure (i.e., the reliable neurons), we also found such neurons that switch categories between the two noises in all auditory structures. However, although we initially found around 40% of such neurons in A1 (Fig. 5*A2*), the bootstrap procedure indicated that more realistic percentages should be much lower, potentially around 20% (Fig. 5*B2*). The response variability, which is probably much larger in awake than in anesthetized animals (Edeline et al., 2000, 2001; Huetz et al., 2009), can potentially explain the difference between our results and those of Ni et al. (2017). Here, these neurons were detected in auditory cortex but were found in higher proportions in the IC and in the auditory thalamus. This indicates that these subcortical neurons might be more sensitive to the sound streams in which the signals were embedded. It is interesting to note that signal-like responses in stationary noise became as much balanced as they remained signal-like in chorus noise. Since in chorus noise, the signal and masker are very close (both spectrally and temporally), this change of category from signal-like to balanced was predictable. As already mentioned by Ni et al. (2017), if a larger number of noise types would have been tested, the proportion of neurons within each category would have been different. For example, a larger fraction of neuronal responses can potentially be considered as signal-like or masker-like, because masker-like responses in a particular type of noise can be the signal-like ones in another noise. Our results show that their assumption, if valid, does not only concern the A1.

Robust perception of speech in humans or vocalizations in animals probably also requires a robust representation of competing sounds (here, masking noise). This can be the functional role of the neurons presenting masker-like responses, which are potentially crucial to determine the characteristics of the noise type and to provide an accurate representation of it within the auditory stream reaching our ears at any time. They were detected here, in higher proportion in the CN in stationary noise, but they became more numerous and in equivalent proportion in all structures in chorus noise. Therefore, the noise representation can be based on the neuronal activity in the CN in stationary noise, whereas this representation can be more distributed in the chorus noise potentially because this noise has more naturalistic temporal properties leading to activate more neurons than the stationary noise.

### Predictors of neuronal behavior in noise

Using classifiers trained with different types of descriptors, we looked for characteristics from the responses to the pure tones (i.e., the TFRPs), to the signal alone and to the noise alone for predicting the assignment of a given recording in a particular category. We pointed out that the TRFP parameters led to an accuracy of the classifier very close to the chance level indicating that the basic static filtering properties derived from the TFRPs did not predict the neuronal behaviors in noise. When adding the descriptors of both the signal alone and noise alone (as the firing rate and the temporal reliability), the classifier reached an accuracy up to around 70% in both noises which strongly suggests that responses to signal alone and to noise alone contained enough information to predict the behavior in noise of a given recording. In a previous study testing the responses of IC cells to vocalizations in noise, it was shown that despite no consistent effect of the mean firing rate, the temporal reliability was decreased by half (Lesica and Grothe, 2008). Under these conditions, IC neurons were still efficient in detecting vocalizations in noise. Using these physiological results in a computational model, this study also pointed out that under noisy conditions, lowpass filtering the noisy vocalizations is the most efficient strategy to code the stimuli because it preserved the power at low modulation frequencies and the temporal reliability of responses.

Together, these results suggest that the more temporally precise are the synaptic inputs converging onto a particular neuron, the more robust is the response of that neuron in background noise. This is true for encoding slow amplitude modulations, which are among the most efficient cues to discriminate speech and communication sounds (Shannon et al., 1995; Zeng et al., 2005; Souffi et al., 2020).

In conclusion, here, we propose that the noise-robustness observed in many studies at the cortical level stems, at least partially, from subcortical mechanisms (Lesica and Grothe, 2008; Lohse et al., 2020). Therefore, the auditory cortex potentially inherits adaptation from earlier levels, allowing the cortical networks to focus on higher-level processing such as classifying the target stimuli into phonetic or linguistic features (Mesgarani et al., 2014), segregating the different auditory streams (Mesgarani and Chang, 2012), integrating multimodal information (Deneux et al., 2019), and retaining behaviorally important stimuli in short term (Huang et al., 2016) or long-term memory (Moczulska et al., 2013; Concina et al., 2019).

## Synthesis

Reviewing Editor: Christine Portfors, Washington State University Decisions are customarily a result of the Reviewing Editor and the peer reviewers coming together and discussing their recommendations until a consensus is reached. When revisions are invited, a fact-based synthesis statement explaining their decision and outlining what is needed to prepare a revision will be listed below. Note: If this manuscript was transferred from JNeurosci and a decision was made to accept the manuscript without peer review, a brief statement to this effect will instead be what is listed below.

Thank you for responding to the previous reviewers' comments in such a thoughtful manner.

Minor edits:

Please start a new paragraph with line 718.

Line 736 - Please describe any differences in results in the VCN versus DCN recordings. I understand it is hard to distinguish the divisions of the CN based on depth and MU recordings but could you speculate. Is there anything obvious in your results that makes you think there may be differences in how VCN and DCN neurons respond to the stimuli?
